# MRI-based contrast clearance analysis shows high differentiation accuracy between radiation-induced reactions and progressive disease after cranial radiotherapy

**DOI:** 10.1016/j.esmoop.2022.100424

**Published:** 2022-03-03

**Authors:** R. Bodensohn, R. Forbrig, S. Quach, J. Reis, A.-L. Boulesteix, U. Mansmann, I. Hadi, D.F. Fleischmann, J. Mücke, A. Holzgreve, N.L. Albert, V. Ruf, M. Dorostkar, S. Corradini, J. Herms, C. Belka, N. Thon, M. Niyazi

**Affiliations:** 1 Department of Radiation Oncology, University Hospital, LMU Munich, Munich, Germany; 2 Institute of Neuroradiology, University Hospital, LMU Munich, Munich, Germany; 3 Department of Neurosurgery, University Hospital, LMU Munich, Munich, Germany; 4 Institute for Medical Information Processing, Biometry and Epidemiology, Faculty of Medicine, LMU Munich, Munich, Germany; 5 German Cancer Consortium (DKTK), Munich, Germany; 6 German Cancer Research Center (DKFZ), Heidelberg, Germany; 7 Department of Nuclear Medicine, University Hospital, LMU Munich, Munich, Germany; 8 Center for Neuropathology and Prion Research, Faculty of Medicine, LMU Munich, Munich, Germany

**Keywords:** pseudoprogression, radiation necrosis, stereotactic radiosurgery, brain metastases, glioma

## Abstract

**Background:**

Pseudoprogression (PsP) or radiation necrosis (RN) may frequently occur after cranial radiotherapy and show a similar imaging pattern compared with progressive disease (PD). We aimed to evaluate the diagnostic accuracy of magnetic resonance imaging-based contrast clearance analysis (CCA) in this clinical setting.

**Patients and methods:**

Patients with equivocal imaging findings after cranial radiotherapy were consecutively included into this monocentric prospective study. CCA was carried out by software-based automated subtraction of imaging features in late versus early T1-weighted sequences after contrast agent application. Two experienced neuroradiologists evaluated CCA with respect to PsP/RN and PD being blinded for histological findings. The radiological assessment was compared with the histopathological results, and its accuracy was calculated statistically.

**Results:**

A total of 33 patients were included; 16 (48.5%) were treated because of a primary brain tumor (BT), and 17 (51.1%) because of a secondary BT. In one patient, CCA was technically infeasible. The accuracy of CCA in predicting the histological result was 0.84 [95% confidence interval (CI) 0.67-0.95; one-sided *P* = 0.051; *n* = 32]. Sensitivity and specificity of CCA were 0.93 (95% CI 0.66-1.00) and 0.78 (95% CI 0.52-0.94), respectively. The accuracy in patients with secondary BTs was 0.94 (95% CI 0.71-1.00) and nonsignificantly higher compared with patients with primary BT with an accuracy of 0.73 (95% CI 0.45-0.92), *P* = 0.16.

**Conclusions:**

In this study, CCA was a highly accurate, easy, and helpful method for distinguishing PsP or RN from PD after cranial radiotherapy, especially in patients with secondary tumors after radiosurgical treatment.

## Highlights

• CCA is accurate in distinguishing treatment reactions from true PD.
• CCA was more accurate for irradiated metastases than primary BTs.
• CCA is not feasible for lesions with no contrast media uptake.

## Introduction

Radiation therapy (RT) is well established as a main pillar in the treatment of a variety of brain tumor (BT) entities. In addition to targeting tumor cells, healthy brain tissue may react to irradiation or concurrent systemic treatment (ST).^1^^,^^2^ In some cases, late reactions known as radiation necrosis (RN) may occur and cause serious adverse effects, which may require antiedematous treatment with steroids or bevacizumab or in advanced cases necrosectomy.^3^^,^^4^ On magnetic resonance imaging (MRI), irradiated lesions may develop new contrast enhancement and perifocal edema;^2^^,^^5^ these radiological characteristics often make this pseudoprogression (PsP) nearly indistinguishable from real progressive disease (PD) on conventional MRI sequences and thus make surgical sampling necessary.^6^

PsP is radiologically defined as progression with increased contrast enhancement, which is not caused by tumor growth. In patients with glioma, PsP occurs mostly within the first 3 months after RT. In patients with brain metastases (BMs) of peripheral tumors, PsP usually occurs at a median period of 7-11 months after RT, but sometimes even after >5 years.^7^, ^8^, ^9^, ^10^ As PsP/RN represents tumor response or a reactive disease state, its distinction from PD has a critical impact on further treatment decisions.^11^

Delayed contrast extravasation MRI as an option for differentiating PD from PsP/RN has initially been described by Zach et al.^12^ in 2015 (2012 with preliminary data^13^). This method uses contrast clearance analysis (CCA) to calculate treatment response assessment maps (TRAMs). Although CCA is the name of the method and TRAM is the associated diagnostic images, both terms are often used in literature as synonyms to describe the same examination. Hereby, the T1-weighted MRI sequences acquired ∼60-105 min [according to instructions given by Brainlab (Munich, Germany)^14^] after the application of gadolinium-based contrast media (CM) are subtracted from those acquired 5 min after the application of CM. After introducing this method in 2015, there was only one recent study which analyzed respective cases but did not correlate them with histological results.^15^

In this study, CCA is evaluated in a prospective controlled setting to examine patients with BM or glioma who underwent cranial RT and present with an equivocal progression at the time of study inclusion, therefore requiring tissue sampling. Independent and blinded neuroradiological reports of the CCA are compared with histopathological results, which were gained after stereotactic biopsy or resection.

## Materials and methods

This study was designed as a prospective study in which patients with equivocal radiological imaging changes after cranial RT were included. The main aim is to independently validate the findings from Zach et al.^12^ to differentiate between tumor recurrence or reactive changes. Patients were included during follow-up examinations after RT. Testing began in May 2019 and ended October 2020; follow-up was completed in January 2021. In this context, apart from the clinical status, any of the following conventional imaging features in follow-up MRI after RT qualified the dedicated brain lesion as (pseudo)progression according to the Response Assessment in Neuro-Oncology (RANO) criteria:^16^^,^^17^ increase of contrast-enhancing lesion by ≥25%; any new contrast-enhancing lesion; and significant increase of perifocal T2/fluid-attenuated inversion recovery non-enhancing lesion on stable or increasing doses of glucocorticosteroids. An interdisciplinary neurooncological tumor board set the indication either for a stereotactic biopsy to differentiate between PD and therapy-related PsP/RN or a resection due to a symptomatic, space-occupying formation.

**Figure 1:**
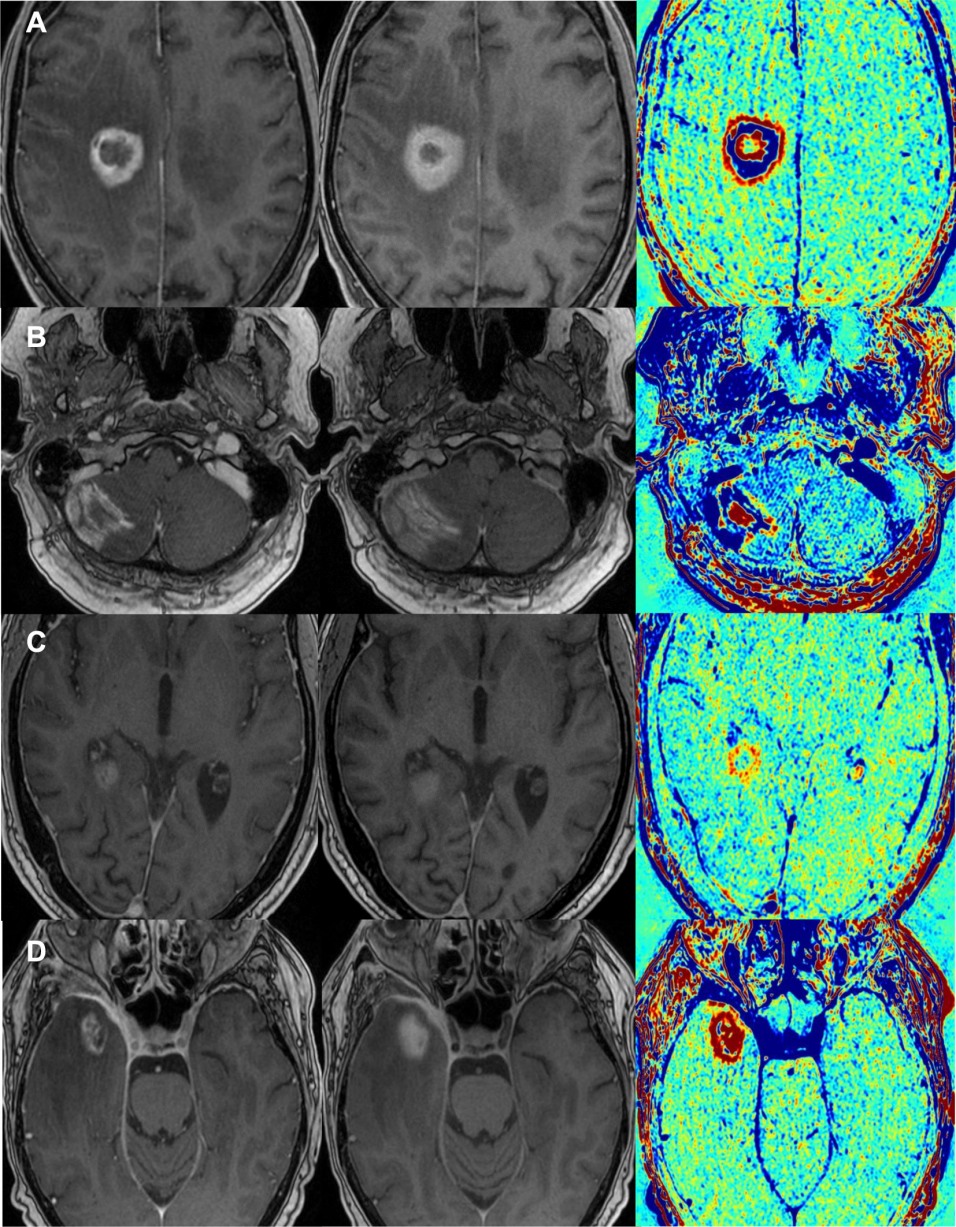
**Examples of contrast clearance analysis (CCA).** The images show four different patients (A-D), each with a regular contrast-enhanced T1-MRI sequence, a late phase T1-sequence ∼1 h after contrast media application, and their CCA (from left to right). Tumor tissue is depicted as blue in the CCA, while reactive tissue is depicted as red. (A) Glioblastoma (WHO 2016 grade IV) IDH wt: a frontoparietal lesion showing tumor tissue in a circular formation with reactive components centrally and at the lesional border (Patient ID 17). (B) Lung adenocarcinoma with brain metastases: a right cerebellar lesion showing tumor tissue with reactive components in the surrounding area (Patient ID 03). (C) Glioblastoma (WHO 2016 grade IV) IDH wt: a periventricular lesion showing spotted areas with reactive tissue (Patient ID 20). (D) Maxillary squamous cell cancer with brain infiltration: a lesion in the right temporal lobe consisting nearly entirely of reactive tissue (Patient ID 25). IDH, isocitrate dehydrogenase; MRI, magnetic resonance imaging; WHO, World Health Organization; wt, wild type.

All MRI examinations were carried out using a 3 Tesla MRI scanner (Signa, General Electric Healthcare, Chicago). The standardized protocol for stereotactic planning consisted of the following sequences: diffusion-weighted imaging (5 mm) in axial plane, spin-echo T2-weighted imaging (2 mm) in axial plane, and 3D T1-weighted imaging before and 5 min after intravenous application of a macrocyclic gadolinium-based contrast agent (Dotagraf, Jenapharm, Jena, Germany) in axial plane including reconstructions in coronal and sagittal planes. The T1-weighted sequence parameters were as follows: fast spoiled gradient echo (FSPGR); repetition time msec/echo time msec, 4.5/1.4; slice thickness, 1 mm with no gap; voxel size, 1 × 1 × 1 mm; matrix, 256 × 256; field of view, 24 cm^2^; flip angle, 15°; and bandwidth, 195 Hz. In addition, the planning MRI was augmented with another (late-phase) T1-weighted sequence after 60 min. Using a dedicated software (Elements, Brainlab, Munich, Germany), CCA was then calculated by automatically subtracting the early from the late-phase T1-weighted dataset as described above. In this analysis, tissue with a rapid washout of the CM is depicted as blue, and tissue with a slow washout is depicted as red. Zach et al.^13^ compared the CCA reaction with histological appearances: regions with rapid washout consisted of dilated lumina, proliferating endothelial cells, and undamaged outline; regions with slow washout, however, presented vessel necrosis with damaged lumina. In theory, progressive lesions are displayed in blue on CCA, as tumor tissue is highly vascularized. As irradiated areas show a leakage of CM due to the radiation-induced vascular damage, reactive tissue is supposedly depicted as red CCA areas. The CCA was visually rated—taking also into consideration the enhancing lesions’ morphology (e.g. nodular or linear) both in the early- and late-phase contrast-enhanced T1-weighted sequences—independently by two experienced neuroradiologists who were blinded concerning the histopathological result. To rate the lesions, the predominant color (blue corresponding to vital tumor versus red corresponding to reactive tissue) was assessed. Examples of different depictions are given in Figure 1. The assessments of the two neuroradiologists were compared, and in case of a deviation, which occurred in one case only, a consensus was formed by both raters. A binary scale was used to rate the results of both histology and CCA (0 = reactive tissue, 1 = tumor tissue).

For further diagnostic evaluation, some patients underwent additional O-(2-^18^F-fluoroethyl)-l-tyrosine positron emission tomography (FET-PET) before surgery, although not required for this particular study. The FET-PET results were assessed in a similar manner by two experienced nuclear medicine specialists blinded to the histological findings.

Tissue samples for histopathological and molecular genetic evaluation were obtained by minimal-invasive stereotactic serial biopsy or open tumor resection. The biopsy procedure was carried out as described in detail before:^18^ In brief, a stereotactic frame (MHT Stereotactic Systems, Freiburg, Germany) was fixed on the patient’s head during general anesthesia. Preoperative MRI and FET-PET data blinded to the results of the CCA were fused to the intraoperatively obtained localized computed tomography. Image fusion and trajectory planning were done using iPLAN Stereotaxy (Brainlab). Biopsy was carried out with an ∼5-mm skin incision and a 3-mm diameter burr hole placed under stereotactic guidance along the planned trajectory. Then, the neurosurgeon decided for targeting the most suspicious contrast-enhancing intralesional areas based on both the contrast-enhanced T1-weighted sequences and blue (and/or red) areas in the CCA. Serial 1-mm^3^ specimens were obtained with a biopsy forceps along the trajectory. If the lesions were symptomatic due to a space-occupying effect (e.g. with a large edema or midline shift), open resection was carried out to stabilize the patient. Tissue sampling in open tumor resection was also guided by MRI (without CCA maps) and (if available) FET-PET imaging data by means of intraoperative neuronavigation (Elements; Brainlab).

Histopathological evaluation was carried out by at least two experienced neuropathologists. Depending on the primary diagnosis, MAP2 (HM-2), GFAP (polyclonal), IDH1(R132H) (polyclonal), p53 (BP53-11), and Ki67 (MIB1) staining was carried out for prediagnosed neuroepithelial tumors, while pancytokeratin (Lu-5), TTF-1 (8G7G3/1), MelanA (MART1; A103), and HMB45 testing was carried out in cases with prior carcinoma or melanoma diagnosis, as appropriate, in addition to routine H&E staining.

General patient and treatment characteristics included sex, age, histology of the primary tumor, prior cranial irradiation, and concomitant or sequential systemic therapy. In addition, the patient’s follow-up data of at least 3 months after therapy was analyzed and compared with the results of both the CCA and biopsy. The follow-up MRIs were again analyzed using the RANO-criteria;^16^^,^^17^ in case of progression, the patients underwent another biopsy or resection. If the result predicted the further course, it was defined as correct for this comparison. Informed consent was obtained from all participants. This study was approved by the Ethics Committee of the University Hospital LMU Munich (Nr. 18-267).

The sample size estimation was as follows: assuming a true accuracy of 90% and setting the significance level to 0.05, 33 patients were required to show an accuracy >70% with a power of at least 80% using a one-sided binomial test (exact power with these parameters: 89%). The accuracy was defined as the probability of CCA predicting the histopathological result correctly. As one patient was not analyzable, a sensitivity analysis was carried out by assuming this patient as either correctly or incorrectly predicted.

## Results

Between May 2019 and October 2020, 33 patients [17 (51.5%) female and 16 (48.5%) male] were prospectively and consecutively recruited. A total of 16 (48.5%) patients were treated for a primary BT, and 17 (51.1%) for a secondary BT, of which 16 (48.5%) had BM of a distant primary (Table 1). Nine (27.3%) patients were on steroid treatment, no patient had received bevacizumab at the time of CCA; 28 (84.8%) patients underwent stereotactic biopsy and 5 (15.2%) patients received a resection. Median time between last RT and CCA was 7.1 months (range 1.2-54.0 months). Median time between contrast-enhanced MRI and MRI for comparison used for the CCA was median 72 min (range 58-89 min).

**Table 1:** An overview of general and treatment-specific patient characteristics

Characteristics	Number of patients	Percentage
Sex (*n* = 33)		
Female	17	51.1
Male	16	48.5
Tumor entities (*n* = 33)		
Primary brain tumors	16	48.5
Glioblastoma (WHO 2016 grade IV) IDH wt	13	39.4
Anaplastic astrocytoma (WHO 2016 grade III) IDH mutation	2	6.1
Low-grade glioma (WHO 2016 grade II)	1	3.0
Secondary brain tumors	17	51.1
Brain metastases	16	48.5
Lung adenocarcinoma	9	27.3
Malignant melanoma	4	12.1
Breast cancer	1	3.0
Rectal cancer	1	3.0
Pleura mesothelioma	1	3.0
Maxillary squamous cell cancer with brain infiltration	1	3.0
Radiation technique of primary radiotherapy (*n* = 33)		
Normofractionated radiotherapy	15	45.5
Moderately hypofractionated radiotherapy	2	6.1
Stereotactic radiosurgery	14	42.4
Hypofractionated stereotactic radiotherapy	1	3.0
Brachytherapy with iodine-125 implants	1	3.0
Radiation technique of reirradiation (*n* = 6)		
Normofractionated radiotherapy	2	33.4
Hypofractionated stereotactic radiotherapy	2	33.4
Whole-brain radiotherapy	1	16.7
Brachytherapy with iodine-125 implants	1	16.7
Systemic treatment during or after primary radiotherapy (*n* = 33)		
Concomitant	15	45.5
Sequential	20	60.6
Systemic treatment during or after reirradiation (*n* = 6)		
Concomitant	1	16.7
Sequential	1	16.7
Method of tissue sampling (*n* = 33)		
Stereotactic biopsy	28	84.8
Resection	5	15.2

IDH, isocitrate dehydrogenase; WHO, World Health Organization; wt, wild type.

Of the patients with metastases, 15 (45.5%) had received stereotactic RT and 1 (3.0%) patient had received brachytherapy with iodine-125 seeds. Of these patients, two were reirradiated, one with whole-brain RT and one with an additional stereotactic RT of a pretreated metastasis. For patients with secondary BTs, median time between last irradiation and CCA was 15.0 months (range 1.2-54.0 months).

Of the patients with primary BTs, 14 had been irradiated with conventional fractionation (30 × 2 Gy) and two with hypofractionation (15 × 2.67 Gy). Four patients with primary BTs were reirradiated: two conventionally fractionated, one hypofractionated, and one received iodine-125 seeds. For patients with primary BTs, median time between last RT session and CCA was 5.4 months (range 1.2-44.1 months), which was significantly lower than for secondary BTs using the Mann-Whitney *U* Test (*P* = 0.02).

Concurrent ST had been administered in 15 (45.5%) cases; sequential ST had been given in 20 (33.3%) cases. During reirradiation (*n* = 6), one (16.7%) patient had received concomitant and one (16.7%) sequential ST.

Using the ordinal scale described in the Methods section, histological results were follows: 15 (45.5%) with 1 = tumor tissue and 18 (54.5%) with 0 = reactive tissue, 10 (62.5%) and 6 (37.5%) for primary BTs, 5 (29.4%) and 12 (70.6%) for secondary BTs, respectively. The CCA results were as follows: 17 (51.5%) with 1 = tumor tissue and 15 (45.5%) with 0 = reactive tissue, 11 (68.8%) and 4 (25.0%) for primary BTs, 6 (35.3%) and 11 (64.7%) for secondary BTs, respectively. In one patient (3.0%) with anaplastic astrocytoma WHO 2016 grade III, CCA evaluation yielded no color-coded information due to missing contrast enhancement of the tumor volume. The patient was not excluded as contrast enhancement was not an inclusion criterion. In 27 (84.4%) cases out of 32 with analyzable CCA, the result of the CCA was identical to the histological result; in five (15.6%) cases CCA showed a discordant result. Imaging examples of patients with PD and PsP are provided in Figure 1. Details about the collected parameters can be found in the Supplementary Material, available at https://doi.org/10.1016/j.esmoop.2022.100424.

The accuracy of CCA in predicting the histology was 0.84 [95% confidence interval (CI) 0.67-0.95] with a nearly significant one-sided *P* value of 0.051 (*n* = 32). The accuracy would be 0.85 (95% CI 0.68-0.95) and the one-sided *P* value 0.041 if the one case, which was not analyzable by CCA, had predicted the histology correctly; if not, accuracy would be 0.82 (95% CI 0.65-0.93) and the one-sided *P* value *P* = 0.09. Sensitivity and specificity of CCA for *n* = 32 were 0.93 (95% CI 0.66-1.00) and 0.78 (95%-CI 0.52-0.94), respectively.

Considering subgroups of primary versus secondary BTs, accuracy was 0.73 and 0.94, respectively; so CCA tended to be more accurate for secondary BTs. This difference, however, was not statistically significant (*P* = 0.16).

When comparing clinical follow-up courses with the histopathological and CCA results, the follow-up result (respective disease state) was correctly predicted in 28 (84.8%; *n* = 33) by histology (whereas surgical results were considered definitive) and in 28 (87.5%; *n* = 32) by CCA. In three (9.4%) cases CCA predicted the correct outcome but histology did not. In two (6.2%) cases CCA was incorrect and histology was correct. In one (3.1%) case, both histology and CCA indicated reactive changes, which was not matched by the later course of disease. Three of five cases falsely predicted by the histological results were glioblastomas, one of them a metastasis of a malignant melanoma and one of them a metastasis of a pulmonal adenocarcinoma. Three of four cases falsely predicted by CCA were glioblastomas and one a metastasis of a pulmonal adenocarcinoma.

A total of 25 patients (75.8%) additionally received an FET-PET examination. For 19 patients, the histological results were predicted correctly, and for 6 patients falsely. The accuracy hereby in predicting the histology was 0.76 (95% CI 0.55-0.91); the result of the FET-PET was not significantly worse than the CCA.

## Discussion

The CCA has been described by Zach et al. in 2015^12^ and was examined by Peker et al.^15^ in early 2021. However, to the best of our knowledge, this study is the first to evaluate CCA in a real-life setting of prospective patients with an equivocal imaging finding on MRI and indication for biopsy/surgery. As a result, CCA was highly accurate (0.84 for *n* = 32, *P* = 0.051) at distinguishing between PD and PsP/RN after RT.

A limitation of the study was that an adequate CCA could not be carried out in one patient and thus the intended case number of 33 patients was not reached. In the respective clinical case of an anaplastic astrocytoma, CCA did not show any output due to missing contrast enhancement, though hypothetically the CCA might have been able to detect small changes of CM uptake after the course of 60 min. However, we generally concluded that CCA is limited in lesions without any CM uptake on diagnostic MRI. Another limitation is the heterogeneous patient cohort, with correspondingly small subgroups. The results already showed a tendency of a better accuracy of CCA for BM but could not prove it due to the small cohort. In addition, histological examination after bioptic tissue sampling is a lot less accurate than after resection as biopsy could miss the region of tumor progression. Concerning the treatment, not all patients received the same kind of RT: higher single fraction doses or re-irradiation has a different biological impact and might lead to a different outcome; nevertheless, the primary risk was similar as the reirradiation was appropriately reduced. All in all, larger cohorts with proper subgroup analysis are needed to answer further questions.

The predictive accuracy remained comparable for 25 patients of this cohort, who additionally received an FET-PET examination. By design, this subcohort was not powered to evaluate FET-PET, but nevertheless the results suggest that CCA might be noninferior compared with FET-PET, which is one of the established methods for the differentiation between PsP and PD.^19^^,^^20^

There were three out of five cases in which the CCA was able to predict the further course of disease correctly in contrast to the histopathological result of the biopsy; therefore even the gold standard is prone to incorrect predictions. It remains speculative whether or not the 1-mm^3^ small tissue samples of a stereotactic biopsy may have been representative of the actual tumor status in these cases. Interestingly, *post hoc* projection of the sampling sites along the biopsy trajectories onto the CCA map confirmed that these areas would have been interpreted as progressive in the CCA. A correlation of biopsy sites and CCA images might give interesting insights in the near future. CCA might be a useful planning tool for not only tissue sampling procedures for stereotactic biopsies but also open tumor resections.

In general, there have been various approaches to differentiate PD from PsP/RN. These approaches mainly refer to patients with glioblastoma. According to the current EANO guidelines, MR perfusion may enable detection of vital BT.^21^ The most commonly applied techniques in the field of BTs are dynamic susceptibility contrast and dynamic contrast-enhanced MR perfusion.^22^ A recent meta-analysis of Chuang and colleagues^23^ yielded high sensitivities and specificities of MR perfusion in the discrimination of tumor recurrence and reactive tissue after RT of (primary and secondary) BTs (ranges 56%-100% and 68%-100%, respectively). However, the meta-analysis yielded very variable cutoff values for an optimal discrimination between both conditions due to heterogeneous methodological designs.^23^^,^^24^ Another approach is the use of magnetic resonance spectroscopy^23^^,^^25^^,^^26^ for which Anbarloui et al.^27^ described a diagnostic accuracy of 81% validated on 33 patients. Akbari et al.^28^ used machine learning with multiparametric MRI and predicted PsP or PD with an overall accuracy of 75% in an interinstitutional validation cohort with 20 patients. Bani-Sadr et al.^29^ suggested that relative cerebral blood volume and relative vessel permeability on MRI-K2 maps could predict early PsP depending on O^6^-methylguanine-DNA methyl-transferase methylation status but did not validate these findings prospectively. The same research group examined conventional MRI radiomics on 76 patients and found an accuracy of 76.0% with a sensitivity of 94.1% and a specificity of 37.5%.^30^ Additionally, non-MRI approaches exist which employ FET-PET for more precise differentiation.^19^ Romagna et al. found sensitivity and specificity of 93% and 84%, respectively.^20^ Finally, there are also nonimaging approaches, such as tumor-educated platelet RNA as a biomarker to distinguish between PD and PsP with a reported accuracy of 85% in a validation cohort (*n* = 20).^31^

Compared with most approaches, CCA has a decisive advantage: it only requires an MRI-scanner, and is, for most patients, an easily available method to distinguish PsP from PD. CCA could delay the need for a biopsy in ambiguous cases or potentially make biopsy unnecessary in the future. In our experience, spatial resolution of CCA may be limited to contrast-enhancing brain lesions >5 mm. In this study, blue lesions in the CCA with a smaller size were not necessarily rated as suspicious for vital tumor tissue (e.g. due to a linear but not nodular morphology in conventional MRI). Furthermore, similar to Peker and colleagues,^15^ we noticed that the lesions’ margins commonly yield color transitions between blue and red (e.g. yellow), disabling clear discrimination of very small findings <5 mm. In patients with such small lesions who underwent only stereotactic biopsy (but not open resection) we therefore recommend continuous MRI follow-up to quickly detect false-negative cases. MR-Perfusion was not routinely carried out in this TRAM study, disabling intermodal comparison of diagnostic accuracy. This comparison is however planned in the future.

### Conclusion

CCA could be a great addition to follow-up MRIs after cranial RT and is better available than, for instance, FET-PET. This method is, however, not feasible for lesions with no CM uptake on diagnostic MRI. Further prospective studies will show how CCA will fit into the diagnostic landscape.
